# NBR1 is dispensable for PARK2-mediated mitophagy regardless of the presence or absence of SQSTM1

**DOI:** 10.1038/cddis.2015.278

**Published:** 2015-10-29

**Authors:** J Shi, G Fung, H Deng, J Zhang, F C Fiesel, W Springer, X Li, H Luo

**Affiliations:** 1 Department of Pathology and Laboratory Medicine, James Hogg Research Center, Providence Heart+Lung Institute, St. Paul's Hospital, University of British Columbia, Vancouver, BC, Canada; 2 Department of Neuroscience, Mayo Clinic, Jacksonville, FL, USA; 3 Mayo Graduate School, Neurobiology of Disease, Jacksonville, FL, USA; 4 Department of Biochemistry and Molecular Biology, Institute of Biomedical Sciences, East China Normal University, Shanghai, China; 5 Department of Molecular and Cellular Biology, Baylor College of Medicine, Houston, TX, USA

## Abstract

Degradation of malfunctional mitochondria by mitophagy is a pivotal component of mitochondrial quality control to maintain cellular homeostasis. Mitochondrial clearance through the PINK1/PARK2 pathway is mediated by autophagic adaptor proteins. Previous studies revealed a significant involvement, but not an absolute requirement for SQSTM1 in PARK2-dependent mitophagy, suggesting that the existence of redundant adaptor proteins may compensate for the loss of SQSTM1. Here we investigated whether NBR1, a functional homolog of SQSTM1, has a role in PARK2-mediated mitophagy, either alone or as a compensatory mechanism. We showed that NBR1 does not appear to be required for mitochondrial clustering following mitochondrial depolarization. Moreover, we demonstrated that deletion of NBR1 alone or in combination with SQSTM1 does not prevent the degradation of damaged mitochondria. Our data suggest that NBR1 is dispensable for PARK2-dependent mitophagy and additional autophagic adaptor proteins, other than NBR1, are responsible for mitochondrial degradation in cells depleted of SQSTM1.

Impaired mitochondrial function has been implicated in a number of human diseases, especially neurodegenerative and heart diseases.^1, 2, 3^ Mitochondria dysfunction not only causes defects in energy generation, but also results in an elevated production of reactive oxygen species and increased apoptosis.^4^ Therefore, mitochondrial quality control (MQC),^5^ a process to preserve functional mitochondria, is extremely important for the maintenance of cellular homeostasis.

MQC depends on a balance between biogenesis and degradation of mitochondria. Efficient clearance of damaged mitochondria by autophagy, termed mitophagy, constitutes a critical component of MQC. Although multiple mechanisms have been suggested to be involved in the regulation of mitophagy,^6, 7, 8, 9^ the PTEN-induced putative kinase 1 (PINK1)/PARK2 pathway is the best characterized and most extensively studied signaling mechanism of mitophagy, partly due to the recognized significance of these two molecules in the pathogenesis of Parkinson's disease.^10, 11, 12^ In healthy non-depolarized mitochondria, the serine/threonine kinase PINK1 is constantly and rapidly turned over by mitochondrial proteases. Upon mitochondrial damage, PINK1 accumulates on the outer membrane of mitochondria and recruits the E3 ubiquitin ligase PARK2/Parkin from the cytosol to depolarized mitochondria, where PARK2 subsequently targets damaged mitochondrial proteins for ubiquitination and bulk degradation by autophagy.^10, 11, 13, 14, 15, 16^

Selective autophagy is mediated by autophagic adaptor proteins.^17^ To date, at least five adaptor proteins, that is, sequestosome 1 (SQSTM1)/p62, neighbor of BRCA1 gene 1 (NBR1), calcium binding and coiled-coil domain 2 (NDP52), TRAF-interacting protein with forkhead-associated domain (T6BP), and optineurin, have been identified.^18, 19, 20, 21, 22^ Among them, SQSTM1 has been widely studied and reported to be involved in PARK2-mediated mitophagy.^16^ Although available data are still controversial, recent evidence supports the notion that SQSTM1 participates in the regulation of mitophagy, yet it is not absolutely required for this process since deletion of SQSTM1 does not block PARK2-dependent disposal of damaged mitochondria. The general assumption is that redundant autophagy receptors, for instance, NBR1, which shares similar functional domains with SQSTM1, have a compensatory role in mitophagy when SQSTM1 is deficient.^23, 24^ However, definitive evidence is lacking.

In the present study, we aim to investigate whether NBR1 has a role in PARK2-dependent mitophagy, either alone or as a compensatory mechanism to overcome SQSTM1 depletion. Following mitochondrial depolarization induced by carbonyl cyanide m-chlorophenylhydrazine (CCCP), a mitochondrial uncoupler, we demonstrated that NBR1 does not appear to be required for mitochondrial clustering. We further showed that deficiency of NBR1 alone or together with SQSTM1 does not block PARK2-dependent clearance of damaged mitochondria. Our results suggest that NBR1 is dispensable for mitophagy regardless of the status of SQSTM1.

## Results

### CCCP treatment induces PARK2-dependent perinuclear clustering and degradation of mitochondrial proteins

**Figure 1:**
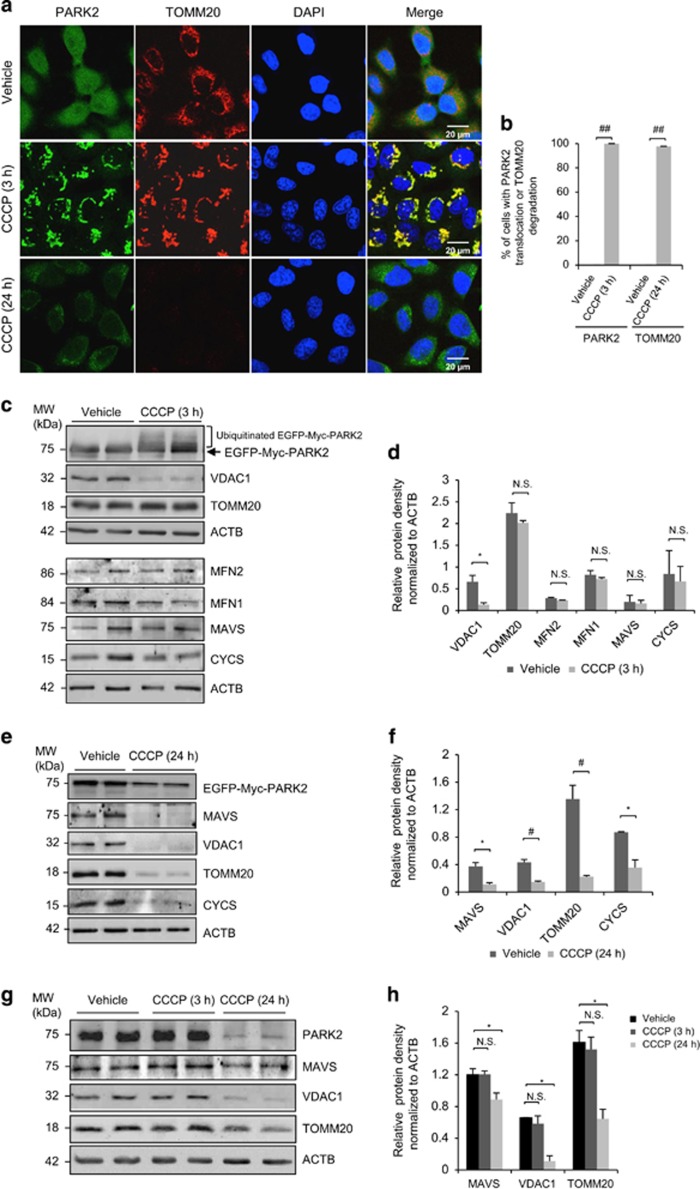
CCCP treatment induces perinuclear aggregation and degradation of mitochondrial proteins in HeLa cells stably expressing PARK2. (**a**, **c**, and **e**) HeLa cells stably expressing EGFP-Myc-PARK2 were treated with either vehicle or CCCP (10 *μ*M) for 3 or 24 h as indicated. PARK2 signal was shown in green. Cells were immunostained for TOMM20 (red). Nuclei were counterstained with DAPI (blue). Western blotting was performed to examine the levels of various mitochondrial proteins as indicated at 3 h (**c**) and 24 h (**e**) post CCCP treatment. ACTB/*β*-actin was probed as a loading control. (**b**) The percentage of cells with PARK2 translocation to mitochondria (left) or TOMM20 degradation (right) relative to total number of cells in (**a**) is presented. Quantification was performed based on three images with each image containing at least 30 cells (mean±standard deviation (S.D.), *n*=3). ^##^*P*<0.001. (**d** and **f**) Densitometric analysis of protein levels after normalized to ACTB in (**c**) and (**e**) (mean±S.D., *n*=3), respectively. *P<0.05; ^#^*P*<0.01; NS, not significant. (**g**) SH-SY5Y cells, a human neuroblastoma cell line with relatively high expression of endogenous PARK2, were treated with vehicle or CCCP (10 *μ*M) for 3 or 24 h as indicated. Western blotting was carried out to examine the protein levels of PARK2, VDAC1, TOMM20, and MAVS. ACTB/*β*-actin was probed as a loading control. (**h**) Densitometric analysis of protein levels of VDAC1, TOMM20, and MAVS after normalized to ACTB (mean±S.D., *n*=3). *P<0.05; NS, not significant

CCCP causes mitochondrial depolarization by increasing membrane permeability to protons and is a widely used mitochondrial uncoupler for the study of mitophagy.^25, 26, 27, 28^ In this study, CCCP was used to investigate the functional role of NBR1 in PARK2-dependent mitophagy. Since regular HeLa cells express low levels of PARK2, HeLa cells stably expressing PARK2 is a widely used tool to delineate the mechanisms of PARK2-dependent mitophagy, and has been shown to response similarly to those cell lines with relatively high levels of exogenous PARK2.^11, 23, 29^ Thus, HeLa cells stably expressing EGFP-Myc-PARK2 were established and used to examine the dynamics of mitochondria after CCCP treatment. The demonstrated features of PARK-mediated mitophagy include the translocation of PARK2 from cytosol to perinulear region and the subsequent degradation of mitochondrial proteins at early and late stages, respectively.^30^ In consistent with previous observations,^25, 26^ we showed that following 3 h of CCCP treatment, PARK2 translocated to the perinuclear regions and co-localized with mitochondrial outer membrane protein TOMM20 (mitochondrial import receptor subunit TOM20 homology) in clusters (Figure 1a, middle panel and b). Ubiquitination of PARK2 was detected after 3 h of CCCP treatment, supporting previous report that PARK2 is self-ubiquitinated in the early stage of mitophagy (Figure 1c).^16^ Furthermore, we found that the expression level of mitochondrial outer membrane protein, voltage-dependent anion channel 1 (VDAC1), but not other mitochondria proteins examined, including TOMM20, mitochondrial antiviral-signaling protein (MAVS), cytochrome C (CYCS), and mitofusion 1 and 2 (data not shown) was markedly reduced after CCCP treatment for 3 h, indicating a specific protein degradation process during early mitochondrial damage (Figures 1c and d). After a prolonged CCCP treatment (24 h),^31, 32, 33^ protein levels of all the tested mitochondrial proteins were dramatically decreased (Figure 1a-bottom panel, B, E, and F), suggesting the bulk clearance of damaged mitochondria. These findings were confirmed using SH-SY5Y cells, a human neuroblastoma cell line with relatively high expression of endogenous PARK2 (Figures 1g and h).

### NBR1 is dispensable for CCCP-induced mitochondrial clustering

**Figure 2:**
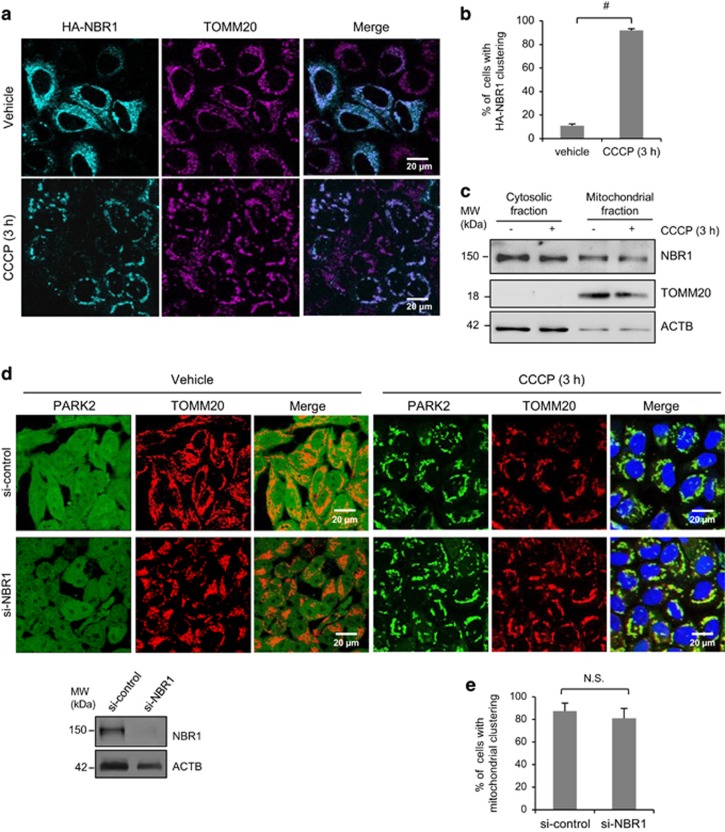
NBR1 is not required for CCCP-induced, PARK2-dependent mitochondrial clustering. (**a**) HeLa cells stably expressing EGFP-Myc-PARK2 were transiently transfected with HA-NBR1 for 24 h, followed by vehicle or CCCP (10 *μ*M) treatment for 3 h. Cells were double immunostained with anti-HA (cyan) and anti-TOMM20 (magenta) antibodies. (**b**) The percentage of cells with HA-NBR1 clustering following 3 h CCCP treatment over total HA-NBR1-positive cells in (**a**) is shown (at least 30 cells were counted for each image, mean±S.D., *n*=3 images). ^#^*P*<0.01. (**c**) HeLa cells stably expressing EGFP-Myc-PARK2 were incubated with vehicle or CCCP (10 *μ*M) for 3 h, followed by the isolation of cytosolic and mitochondrial fractions. Western blotting was performed to examine cellular distribution of NBR1 under basal and CCCP-stimulated conditions. TOMM20 and ACTB/*β*-actin were used as mitochondrial and cytosolic markers, respectively. The images are representative of two independent experiments. (**d**) PARK2 stably expressing HeLa cells were transiently transfected with control siRNA (si-control) or siRNA against *NBR1* (si-*NBR1*) for 48 h, followed by vehicle or CCCP (10 *μ*M) treatment for 3 h. Cells were immunostained with anti-TOMM20 antibody (red). Nuclei were counterstained with DAPI (blue). The si-NBR1 knockdown efficiency was assessed by western blotting using anti-NBR1 antibody. ACTB/*β*-actin was probed as a loading control. (**e**) Immunostaining results in (**d**) were quantified as percentages of cells with mitochondrial clustering over total number of cells (at least 30 cells were counted for each image). Results are presented as mean±S.D., *n*=3 images. NS, not significant

It has been previously reported that SQSTM1 is required for PARK2-dependent mitochondrial perinuclear clustering triggered by CCCP treatment.^23, 34^ We therefore questioned whether NBR1, a functional homolog of SQSTM1, has a similar role. HeLa cells stably expressing PARK2 were transiently transfected with HA-tagged NBR1 for 24 h, followed by vehicle or CCCP (10 *μ*M) treatment for 3 h. As shown in Figures 2a and b, in vehicle-treated cells NBR1 appeared to be co-localized with TOMM20. Upon CCCP treatment, mitochondria were depolarized as demonstrated by perinuclear clustering of TOMM20. Figure 2a also showed that NBR1 was co-localized with depolarized mitochondria in CCCP-treated cells, implying a relationship between NBR1 and mitochondria. To determine whether NBR1 is associated with mitochondria, cell fractionation was conducted. Figure 2c revealed that NBR1 was present in both cytosolic and mitochondrial fractions under normal condition. Treatment with CCCP did not seem to alter the distribution of NBR1. To determine the definitive function of NBR1 in mitochondrial clustering, NBR1 in PARK2 stable HeLa cells was knocked down using siRNA technique. Confocal microscopy showed that gene silencing of NBR1 had essentially no effect on CCCP-induced mitochondrial translocation of PARK2 and formation of mitochondrial aggregates (Figures 2d and e). Together, our data suggest that NBR1 is not a necessary mediator in mitochondrial clustering.

### PARK2-dependent degradation of VDAC1 is mediated through the proteasome pathway

**Figure 3:**
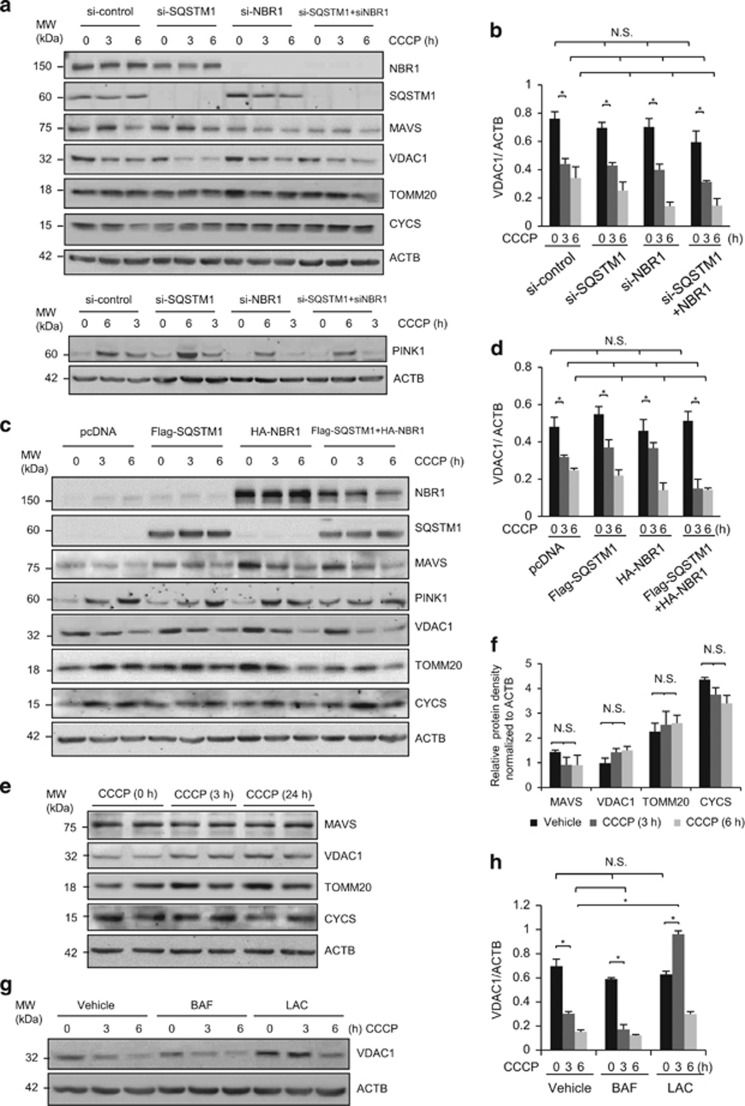
PARK2-dependent early degradation of VDAC1 is mediated through the ubiquitin-proteasome pathway. (**a**) HeLa cells stably expressing PARK2 were transiently transfected with si-*NBR1* and si-*SQSTM1* alone or in combination as indicated for 48 h, followed by vehicle or CCCP (10 *μ*M) treatment for 3 or 6 h. Cells transfected with si-control were used as controls. Western blotting was performed to examine protein expression of NBR1, SQSTM1, and various mitochondrial proteins as indicated. (**b**) Densitometric analysis of VDAC1 after normalized to ACTB in (**a**) (mean±S.D., *n*=3). *P<0.05; NS, not significant. (**c**) PARK2 stably expressing HeLa cells was transiently transfected with HA-NBR1 and Flag-SQSTM1 alone or in combination as indicated for 24 h, followed by vehicle or CCCP (10 *μ*M) treatment for 3 or 6 h. Cells transfected with empty vector were used as controls. Protein levels of NBR1 (using anti-NBR1 antibody), SQSTM1 (using anti-FLAG antibody), and various mitochondrial proteins were examined as described above. (**d**) Densitometric analysis of VDAC1 after normalized to ACTB in (**c**) (mean±S.D., *n*=3). *P<0.05; NS, not significant. (**e**) Regular HeLa cells were treated with CCCP (10 *μ*M) for 3 h or 24 h. Western blot analysis was carried out to examine the expression of various mitochondrial proteins as indicated. (**f**) Densitometric analysis of protein levels after normalized to ACTB in (**e**) (mean±S.D., *n*=3). NS, not significant. (**g**) HeLa cells stably expressing PARK2 were treated with CCCP (10 *μ*M) for indicated time periods in the presence or absence of lysosomal inhibitor bafilomycin A1 (BAF, 200 nM) or proteasome inhibitor lactacystin (LAC, 10 *μ*M). Protein level of VDAC1 was examined by western blotting. (**h**) Densitometric analysis of protein levels after normalized to ACTB in (**g**) (mean±S.D., *n*=3). *P<0.05; NS, not significant

We next determined whether NBR1 has a role in CCCP-triggered early downregulation of VDAC1 (Figure 1c). The results presented in Figures 3a–d demonstrated that either knockdown or overexpression of NBR1 alone or in combination with SQSTM1 had no significant impact on CCCP-induced reduction of VDAC1 protein level. Furthermore, we showed that CCCP-induced accumulation of PINK1 is not influenced by modulation of SQSTM1 and/or NBR1 (Figures 3a and c).

To understand whether PARK2 is required for decreased VDAC1 expression following CCCP treatment, regular HeLa cells that do not express PARK2 were treated with CCCP for 3 or 24 h. As shown in Figures 3e and f, protein levels of VDAC1 remained unchanged after CCCP treatment, suggesting that downregulation of VDAC1 is PARK2 dependent. To further explore the underlying mechanism of VDAC1 degradation, PARK2 stable HeLa cells were treated with either proteasome inhibitor lactacystin or lysosome inhibitor bafilomycin A1. We found that addition of lactacystin, but not bafilomycin, inhibited CCCP-induced downregulation of VDAC1, indicating a proteasome-dependent mechanism of VDAC1 degradation (Figures 3g and h). Taken together, our results suggest that CCCP treatment induces PARK2-dependent VDAC1 degradation through the ubiquitin-proteasome pathway. Both NBR1 and SQSTM1 do not appear to be involved in this process.

### NBR1 is dispensable for CCCP-induced mitophagy regardless of the presence or absence of SQSTM1

**Figure 4:**
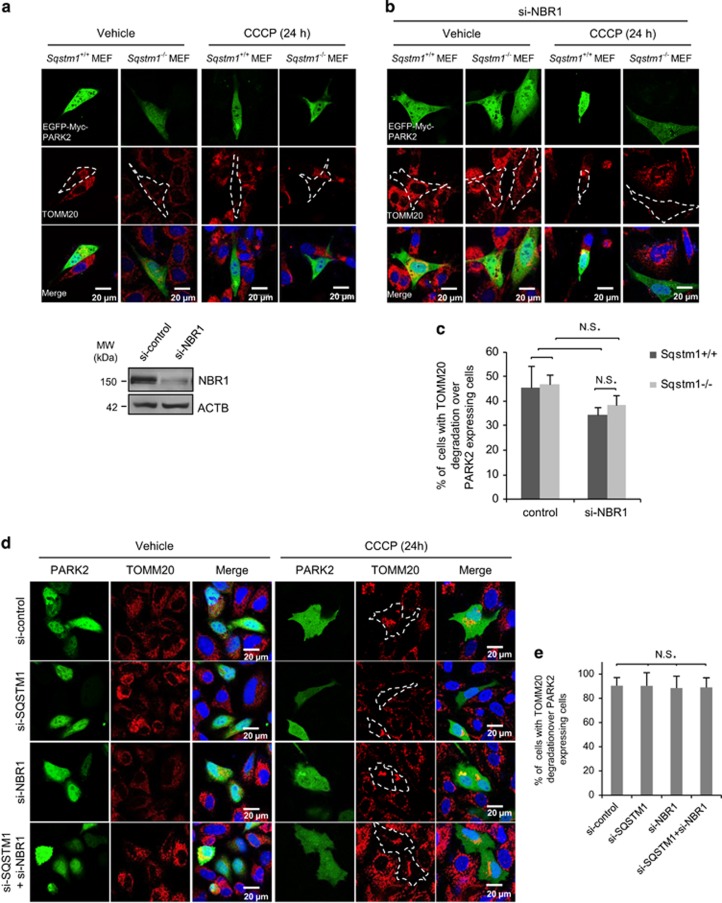

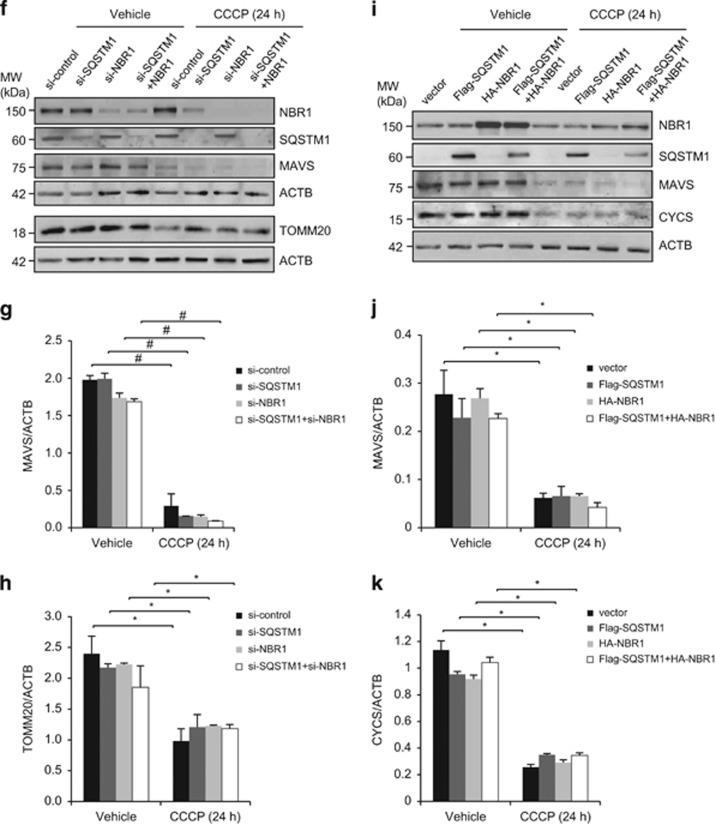
NBR1 is dispensable for CCCP-induced mitophagy regardless of the presence or absence of SQSTM1. (**a**) S*qstm1* wide-type (*Sqstm1*^*+/+*^) or deficient (*Sqstm1*^*−/−*^) MEFs were transiently transfected with a plasmid expressing EGFP-Myc-PARK2 for 24 h. Cells were then treated with vehicle or CCCP (10 *μ*M) for 24 h. PARK2 was displayed in green and TOMM20 was stained in red. The percentage of cells with TOMM20 degradation relative to total counted cells expressing PARK2 (*n*) was presented. (**b** and **d**) *Sqstm1* wide-type or deficient MEFs (**b**) and regular HeLa cells (**d**) were transiently transfected with si-*NBR1* for 24 h, followed by second transfection with EGFP-Myc-PARK2 plasmid for additional 24 h. Cells were then treated with vehicle or CCCP for 24 h. PARK2 signal was shown in green and TOMM20 was stained in red. Quantification was performed as described above. The si-NBR1 knockdown efficiency in MEFs was evaluated by western blotting. (**c** and **e**) Immunostaining results in (**a**, **b**, and **d**) were quantified as percentages of cells with TOMM20 degradation relative to total number of PARK2-expressing cells (at least 10 cells were counted for each image in **a** and **b** and 30 or more cells were counted for each image in **d**, mean±S.D., *n*=3 images). NS, not significant. (**f**) PARK2 stably expressing HeLa cells were transiently transfected with si-*NBR1* and si-*SQSTM1* alone or in combination as indicated for 48 h, followed by vehicle or CCCP (10 *μ*M) treatment for 24 h. The knockdown efficiency of si-*NBR1* and si-*SQSTM1* and protein levels of mitochondrial proteins were examined by western blotting. (**g** and **h**) Densitometric analysis of protein levels of MAVS and TOMM20 after normalized to ACTB (mean±S.D., *n*=3). ^#^*P*<0.01; *P<0.05. (**i**) HeLa cells stably expressing PARK2 were transiently transfected with HA-NBR1 and Flag-SQSTM1 alone or in combination as indicated for 24 h, followed by vehicle or CCCP (10 *μ*M) treatment for 24 h. The cDNA transfection efficiency and protein levels of mitochondrial proteins were examined by western blotting. (**j** and **k**) Densitometric analysis of protein levels of MAVS and CYCS after normalized to ACTB (mean±S.D., *n*=3). *P<0.05

Finally, we examined the role of NBR1 in CCCP-induced mitophagy. As mentioned earlier, the function of SQSTM1 in regulating mitophagy is still controversial. Here we first utilized the *Sqstm*1^*−/−*^ MEFs to evaluate the effects of depletion of SQSTM1 on CCCP-induced mitophagy. Since MEFs express very low levels of PARK2, we transiently transfected exogenous PARK2 into these cells, followed by CCCP treatment for 24 h. Confocal microscopy data showed that TOMM20 was easily detected in vehicle-treated cells. In contrast, CCCP-treated cells display little to no TOMM20 fluorescent signal in PARK2 expressing cells independent of SQSTM1 (Figure 4a). These data indicate that SQSTM1 is not an essential modulator in the removal of damaged mitochondria, in agreement with previous reports.^23, 34^

We next examined the role of NBR1 in mitophagy in the presence or absence of SQSTM1. As shown in Figure 4b, following CCCP treatment, TOMM20 signal was undetectable in PARK2-positive MEFs with either knockdown of NBR1 alone (*Sqstm*1^*+/+*^ MEFs+si-*Nbr1*) or in combination with SQSTM1 (*Sqstm*1^*−/−*^ MEFs+si-*Nbr1*). Statistical analysis revealed no significant difference in the number of cells with TOMM20 degradation in the presence or absence of NBR1 in PARK2 expressing, *Sqstm*1^*+/+*^ and *Sqstm*1^*−/−*^ MEFs (Figure 4c). Similar results were obtained using regular HeLa cells with transient transfection of PARK2, together with si-*NBR1* alone or in combination with si-*SQSTM1* (Figures 4d and e). Since CCCP treatment in HeLa cells stably expressing PARK2 resulted in undetectable levels of TOMM20 throughout the entire field as shown in Figure 1a bottom panel, we chose to utilize HeLa cells transiently transfected with PARK2 so that confocal microscopic images can identify both PARK2-positive and PARK2-negative cells in a single field. In this way, CCCP-treated cells that fail to express PARK2 will exhibit detectable levels of TOMM20, demonstrating a drastic contrast of TOMM20 degradation in cells with PARK2 expression. It is noted that mitochondrial proteins were not completely cleared following 24 h CCCP treatment in PARK2 expressing HeLa cells (Figure 4d). This is likely due to a high cell density, which results in decreased sensitivity to CCCP treatment. Consistent with the immunofluorescence results, western blot analysis demonstrated that neither knockdown nor overexpression of NBR1 alone or in combination with SQSTM1 has significant impacts on CCCP-induced degradation of mitochondrial proteins (Figures 4f–k). Collectively, our results suggest that NBR1 is dispensable for the final removal of damaged mitochondria regardless of the presence or absence of SQSTM1.

## Discussion

Selective removal of terminally damaged organelles that are highly toxic to the cells through autophagy represents an important self-defense mechanism to protect against cell damage. Autophagy adaptor proteins, including SQSTM1 and NBR1, have been revealed to be essential in mediating selective autophagy.^20, 21, 35, 36, 37^ SQSTM1 and NBR1 share the similar functional domains, including the Phox/Bem1p (PB1) domain, the microtubule-associated protein 1 light chain 3 alpha (LC3)-interacting regions (LIR), and the ubiquitin association (UBA) domain.^38^ Through interacting with both LC3 and ubiquitin chains, they function as autophagy receptors targeting ubiquitinated proteins/organelles to autophagosomes for degradation. Functional redundancy of these two proteins has been proposed in the clearance of ubiquitinated proteins^21^ and damaged mitochondria;^23, 24^ however, direct experimental evidence is still missing.

The major findings of the current study are two-fold. First, NBR1 alone does not seem to have a key role in PARK2-mediated mitophagy as neither knockdown nor overexpression of NBR1 affects CCCP-induced mitochondrial clearance. Second, NBR1 does not appear to serve as a compensatory mechanism for the loss of function of SQSTM1 in regulating mitophagy as deletion of both SQSTM1 and NBR1 fails to rescue mitochondrial degradation triggered by CCCP. Our results in this study suggest that additional autophagic adaptor proteins are responsible for the regulation of PARK2-dependent mitophagy. Alternatively, depletion of SQSTM1 results in the activation and recruitment of redundant proteins other than NBR1 to dysfunctional mitochondria. It was previously shown that outer mitochondrial membrane protein BCL2/adenovirus E1B 19kDa interacting protein 3-like (Nix/BNIP3)^26^ and FUN14 domain containing 1 (FUNDC1)^39^ act as a receptor for CCCP and hypoxia-induced mitophagy, respectively. Moreover, it was recently reported that upon depolarization or oxidative stress, optineurin is recruited to depolarized mitochondria to facilitate mitochondrial degradation by autophagy.^40^ Most recently, autophagy/beclin-1 regulator 1 (AMBRA1) was identified as a novel autophagy receptor for impaired mitochondria through binding to autophagosome LC3 in both PARK2- and SQSTM1-dependent and independent manners.^41^ Whether these proteins have a compensatory role in mitophagy in SQSTM1 and NBR1-deficient cells warrants further investigation.

In this study, we demonstrated that NBR1 is co-localized with the depolarized mitochondria although it may not be a necessary mediator for mitophagy. Together with early reports that SQSTM1 co-localizes with mitochondria marker TOMM20 and PARK2 upon stress,^16, 23, 34^ our results suggest that both NBR1 and SQSTM1 are likely targets of mitophagy themselves. This hypothesis was supported by the data presented in Figures 4f and i that protein levels of NBR1 and SQSTM1 are markedly decreased in CCCP-treated cells as compared with control.

Another interesting observation of this study is PARK2-dependent selective proteasomal degradation of VDAC1 at the early stage of mitochondrial damage. This result is consistent with a previous report that degradation of VDAC1 is slowed down, although not completely prevented, with proteasome inhibitors MG132 and epoxomicin.^42^ Additionally, it has been observed that other mitochondrial proteins, such as TOMM20, TOMM40, TOMM70, Omp25, and mitofusins, undergo proteasomal degradation before the entire damaged mitochondria are destined for autophagic degradation.^25, 43, 44^ These findings point to important cellular strategies to maintain a functional network of mitochondria at different phases of organ damage. Here we propose a model of the collaborative function of the proteasome and autophagy pathways in the control of mitochondrial quality during different stages of damage. During the early stage of mitochondrial damage, the proteasome pathway has a key role in the quality control through disposal of individual damaged proteins. However, an extended injury results in a massive accumulation of misfolded proteins that exceed the proteolytic capacity of the proteasome.^45, 46^ Under this condition, the general autophagy and mitophagy pathways are therefore activated to remove the misfolded proteins and the damaged parts of mitochondria from the cells to restore homeostasis.

In summary, our results reveal that NBR1 is neither an essential protein in PARK2-mediated mitophagy nor does it have a major compensatory role in mitochondrial degradation in SQSTM1-depleted cells.

## Materials and Methods

### Cell culture and chemicals

HeLa cells stably expressing EGFP-Myc-PARK2 that was established as described previously,^47^ regular HeLa cells (American Type Culture Collection, ATCC CCL-2), and SH-SY5Y cells (ATCC CRL-2266) were cultured in Dulbecco's modified Eagle's medium with 10% fetal bovine serum, 100 *μ*g/ml penicillin, and 100 *μ*g/ml streptomycin. *sqstm1*^*−/−*^ MEFs (a generous gift from Dr. Masaaki Komatsu, Tokyo Metropolitan Institute of Medical Science, Japan) were maintained in Dulbecco's modified Eagle's medium supplemented with 10% fetal bovine serum, 100 *μ*g/ml penicillin, 100 *μ*g/ml streptomycin, 1 × non-essential amino acids (Life Technologies, Burlington, ON, Canada; 11140-050), and 1 × sodium pyruvate (Life Technologies, 11360-070) at 32.5 °C, supplied with 5% CO_2_. Carbonyl cyanide m-chlorophenylhydrazone (CCCP) was purchased from Santa Cruz Biotechnology (Dallas, TX, USA; sc-202984). Bafilomycin A1 (BAF) and lactacystin (LAC) were purchased from LC Laboratories (Woburn, MA, USA; B-1080) and Sigma-Aldrich (Oakville, ON, Canada; L6785), respectively.

### Plasmids, siRNAs, and transient transfection

The EGFP-Myc-PARK2 plasmid was established as previously described.^47^ The Flag-tagged SQSTM1 and HA-NBR1 constructs were kindly provided by Dr. Brett Finlay (University of British Columbia, Canada) and Dr. Caroline Whitehouse (King's College London, United Kingdom), respectively. The small interfering RNA (siRNA) against *SQSTM1* (human) and *NBR1* (human) was purchased from Dharmacon (Lafayette, CO, USA; M-010230-00-0005 and L-010522-00-0005). si-*Nbr1* (mouse) was purchased from Santa Cruz Biotechnology (sc-149849). cDNA or siRNA transfections were performed using Lipofectamine 2000 (Life Technologies, 11668-019) for 24–48 h according to the manufacturer's protocol.

### Western blot analysis

Cells were washed in cold Dulbecco's phosphate-buffered saline (DPBS) and lysed in Modified Oncogene Science lysis buffer (MOSLB) (50 mM NaPyrophosphate, 50 mM NaF, 50 mM NaCl, 5 mM EDTA, 5 mM EGTA, 100 *μ*M Na_3_VO_4_, 10 mM HEPES, and 0.1%Triton X-100). Protein concentration was measured by either Bradford assay (Bio-Rad Laboratories, Hercules, CA, USA; #500-0006) or BCA protein assay (Thermo Scientific, Rockford, IL, USA; 23227). SDS-PAGE was carried out with 20–40 *μ*g of protein loading. Western blotting was performed according to standard protocol as previously described. The antibodies used in this study were anti-*SQSTM1* (PROGEN Biotechnik GmbH, Heidelberg, Germany; GP62-C), anti-cytochrome c (Santa Cruz Biotechnology, H-104, sc-7159), anti-TOMM20 (Santa Cruz Biotechnology, FL-145, sc-11415), anti-NBR1 (Santa Cruz Biotechnology, sc-130380), anti-ACTB/*β*-actin (Sigma, Oakville, ON, Canada; A5316), anti-HA (Roche, Mississauga, ON, Canada; 11867423001), anti-PARK2 (Cell Signaling, Danvers, MA, USA; Prk8, #4211), anti-VDAC1 (Cell Signaling; #4866), and anti-MAVS (Cell Signaling, #8348), and anti-PINK1 (GeneTex, N3C3, Irvine, CA, USA; GTX107851). Protein levels were quantitated by densitometric analysis using NIH ImageJ software (National Institutes of Health, Bethesda, MD, USA; http://rsb.info.nih.gov/ij/) and normalized to those of ACTB.

### Immunofluorescence

Cells were washed in cold DPBS and fixed with 4% formaldehyde for 30 min at room temperature. After fixation, cells were permeabilized with 0.5% Triton X-100 in DPBS for 10 min, and blocked with 5% bovine serum album in Tris-buffered saline plus Tween 20 (TBS-T) for 30 min. Primary antibodies (anti-HA or anti-TOMM20, diluted 1 : 200) were incubated at 4 °C overnight and secondary antibodies were incubated for 1 h at room temperature. Coverslips were washed with TSB-T, followed by counterstaining with DAPI (4,6-diamidino-2-phenylindole, Vector Laboratories, Burlington, ON, Canada; H-1200). Leica SP2 AOBS confocal fluorescence microscope was employed to obtain high-resolution images. The quantification of the confocal images was conducted by counting at least three images where each image contained 30 or more cells unless otherwise specified.

### Isolation of cytosolic and mitochondrial fractions

Cell fractionation was performed using a mitochondria isolation kit (Pierce Biotechnology, Rockford, IL, USA; PI-89874). Briefly, cells were scraped in DPBS and centrifuged at 850 × *g* for 2 min. Following the addition of reagent A provided by the kit, cells were mixed by vortex and incubated on ice for 2 min. After incubation, cells were transferred to a tissue grinder and homogenized. Reagent C was added to the homogenized lysates and centrifugation was performed at 700 × *g* for 10 min. The supernatant from first centrifugation was further centrifuged at 3000 × *g* for 15 min. The pellet and the supernatant from the second centrifugation containing intact mitochondrial and cytosolic proteins, respectively, were used for western blot analysis.

### Statistical analysis

All data presented are representative of at least three independent experiments. Results are presented as mean±standard deviation (S.D.). Statistical analysis was performed with unpaired, two-sided Student's *t*-test. The probability value of <0.05 was considered to be statistically significant.

## Abbreviations

ACTB: actin, beta
BAF: Bafilomycin A1
CCCP: carbonyl cyanide m-chlorophenylhydrazine
DAPI: 4, 6-diamidino-2-phenylindole
DPBS: Dulbecco's phosphate-buffered saline
LAC: lactocystin
LC3: microtubule-associated protein 1 light chain 3 alpha
MAVS: mitochondrial antiviral-signaling protein
MEFs: mouse embryonic fibroblasts
MQC: mitochondrial quality control
NBR1: neighbor of BRCA1 gene 1
PINK1: PTEN-induced putative kinase 1
si-*NBR1*: *NBR1* siRNA
si-*SQSTM1*: *SQSTM1* siRNA
SQSTM1/p62: sequestosome 1
TOMM20: mitochondrial import receptor subunit TOM20 homology
VDAC1: voltage-dependent anion channel 1
